# Outpatient monitoring of patients with multisystem inflammatory syndrome (MIS-C): A mini review

**DOI:** 10.3389/fped.2022.1069632

**Published:** 2022-12-07

**Authors:** Jerin Tresa Jose, Elif Seda Selamet Tierney

**Affiliations:** Division of Pediatric Cardiology, Lucile Packard Children's Hospital, Stanford University Medical Center, Palo Alto, CA, United States

**Keywords:** MIS-C, COVID-19, follow-up, echocardiography, outpatient MIS-C: multisystem inflammatory syndrome in children, LVEF: left ventricular ejection fraction, echo: echocardiography, coronary artery dilation

## Abstract

**Introduction:**

As we learn more about the novel multisystem inflammatory syndrome in children (MIS-C) associated with COVID-19 infection, the protocols for long-term follow-up have evolved and only some of these protocols have been published. Here, we review the current literature on follow-up guidelines in MIS-C patients.

**Methods:**

We conducted a PUBMED search of all articles published on “MIS-C” and the term “follow-up” between 2020 and 2022. Inclusion criteria were that (1) the study was an observational study or case series, and (2) the study population included pediatric population who met the diagnostic criteria for MIS-C.

**Results:**

There were 206 publications on MIS-C and follow-up in the last 2 years with 11 studies that fit the inclusion criteria. These papers were representing 11 different centers and encompassed a total of 343 participants. Seven of the 11 studies had participants follow-up with their cardiologist within 1 month of discharge. Between 12% and 62% of patients within each study had depressed left ventricular ejection fraction (LVEF) at admission. At the initial follow-up visit, five studies showed a normal LVEF in all patients while the other seven studies showed 2%–13% patients continuing to have depressed LVEF. In eight of the 11 studies, 9%–52% of patients had coronary artery dilation at admission. At their initial follow-up visit, 3%–28% of patients continued to have coronary artery dilation.

**Conclusion:**

There is some institutional variation in the outpatient follow-up protocols in patients diagnosed with MIS-C. A standardized follow-up guidelines might be helpful to monitor long-term prognosis of these patients.

## Introduction

Our understanding of multi-inflammatory syndrome in children (MIS-C) associated with COVID-19 infection has grown significantly over the course of the global pandemic. There are many published studies describing the cardiac involvement of MIS-C with elevated markers of myocardial damage (troponin, B-type natriuretic peptide, etc.), ventricular dysfunction, coronary artery dilation, and arrhythmias (1). Many children with MIS-C present with hypotension and shock requiring admission to intensive care unit and have prolonged hospital courses. As these children are discharged, recommendations regarding follow-up visits with pediatric cardiology and timing of echocardiography remains largely institution-dependent with many institutions adopting timelines similar to Kawasaki Disease or myocarditis guidelines (1, 2). Current CDC and AAP guidelines remain largely broad with recommendation to follow-up with pediatric cardiology 2–3 weeks after discharge (3, 4). There are currently several published studies from multiple institutions on their MIS-C population with follow-up cardiology visits and echocardiography results. The purpose of our study is to review the current published data on the cardiology follow up guidelines for MIS-C patients, along with their echocardiography results, to understand the variability in current practice.

## Methods

We conducted a PUBMED search with keywords “MIS-C” and “Follow-up” between 2020 and 2022. We selected case series and observational studies that fit our inclusion and exclusion criteria. Studies which met the following criteria were included: (1) the study design was an observational study or case series, (2) the study population included pediatric population (<21 years old) who met the diagnostic criteria for MIS-C as established by Centers for Disease Control and Prevention (4). The following information was extracted: author(s), year of publication, sample size, admission and follow-up echocardiography findings, and timing of follow-up pediatric cardiology visits.

## Results

### Yield of the search

**Figure 1:**
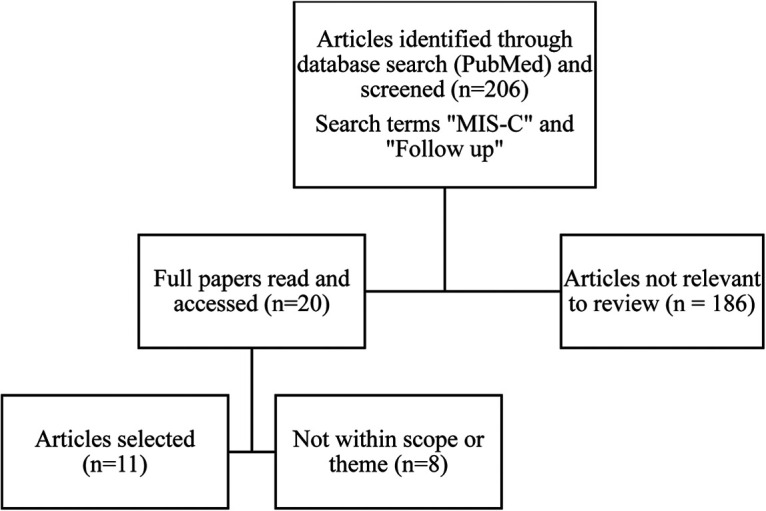
PubMed selection process.

There were 206 publications that resulted with our keyword search on MIS-C and follow-up between 2020 and 2022. Eleven published studies fit the inclusion criteria with a total of 343 participants (5–15). All studies included patients who met criteria for MIS-C per the CDC guidelines (see Figure 1).

### First follow-up timeline

We summarize the follow-up timeline of the included studies in Table 1. Seven of the 11 studies had participants follow-up with pediatric cardiology within 1 month of discharge regardless of the hospital course or echocardiography findings during hospital admission. In the other four studies there was variation. Sirico et al. and Chakraborty et al. had patients follow-up within 2 months, Patnaik et al. had patients follow-up at 3–4 months, and Arslan et al. had patients follow-up at 6 months.

**Table 1:** Follow-up timelines from the included studies.

Author	*n*	Age cutoff	Inclusion criteria	*n*	1st follow-up	*n*	2nd follow-up	*n*	3rd follow-up	*n*	4th follow-up
Farooqui et al.	45	<21 years	MIS-C	39	1–4 weeks	31	1–4 months	24	4–9 months	-	-
Capone et al.	50	<21 years	MIS-C	47	2 weeks	42	8 weeks	24	6 months	-	-
Das et al.	36		MIS-C	36	2–4 weeks	36	2 months	-	-	-	-
Chakraborty et al.	21	<21 years	MIS-C	21	6 weeks	21	6 months	-	-	-	-
Arslan et al.	34	<21 years	MIS-C	17	6 months	-	-	-	-	-	-
Barris et al.	16	<21 years	MIS-C	16	1 month	16	7 months	-	-	-	-
Garbin et al.	32		MIS-C	32	1 week	32	6 months	-	-	-	-
Jhaveri et al.	15	<21 years	MIS-C, at least one echocardiography	13	1 month	-	-	-	-	-	-
Omeir et al.	41	<18 years	MIS-C	38	1 month	29	3 months	28	6 months	7	12 months
Patnaik et al.	21	<19 years	MIS-C	16	3–4 months	-	-	-	-	-	-
Sirico et al.	32		MIS-C	28	2 months	-	-	-	-	-	-

### Second or further follow-up timeline

Four of these 11 studies did not have data regarding further follow-up visits. Of the seven studies that had second follow-up visit data, the timeline varied between 2 and 6 months. Only three of the 11 studies had data about a third follow-up visit which was around 6 months. Two studies had fourth follow-up visit data which was 1 year from discharge.

### Echocardiography results

We only extracted LV function and coronary dilation data for this review due to limited information available from the included publications.

Table 2 summarizes the left ventricular function assessment by ejection fraction (LVEF) results from admission and follow-up that are available from each study being reviewed. In terms of grading the left ventricular systolic function, four of the 11 studies categorized left ventricular function data into normal, mild, moderate, and severe. The other seven studies provided data as normal vs. depressed systolic function. The percentage of patients with depressed LVEF at admission ranged between 12% and 62%.

**Table 2:** Left ventricular ejection fraction (LVEF) data.

	Left ventricular ejection fraction (LVEF) definition	Admission	1st follow-up	2nd follow-up	3rd follow-up	4th follow-up
Farooqui et al.	Normal (LVEF >50%)	51%	90%	97%	100%	-
	Mild	18%	8%	3%	0%	-
	Moderate or severe	7%	0%	0%	0%	-
Capone et al.	Mild (LVEF 45%–55%)	30%	2%	0%	0%	-
	Moderate (LVEF 35%–45%)	22%	0%	0%	0%	-
	Severe (LVEF <35%)	0%	0%	0%	0%	-
Das et al.	Mild	42%	10%	0%	-	-
	Moderate	0%	0%	0%	-	-
	Severe	0%	0%	0%	-	-
Chakraborty et al.	Normal (LVEF >55%)	24%	100%	100%	-	-
	Mild (LVEF 45%–54%)	43%	0%	0%	-	-
	Moderate (LVEF 35–44%)	24%	0%	0%	-	-
	Severe (LVEF <35%)	10%	0%	0%	-	-
Arslan et al.	Depressed	12%	0%	-	-	-
Barris et al.	LVEF <55%	44%	13%	0%	-	-
Garbin et al.	LVEF <45%	31%	0%	0%	-	-
Jhaveri et al.	LVEF <55%	62%	23%	-	-	-
Omeir et al.	LVEF <50%	12%	0%	0%	0%	0%
Patnaik et al.	LVEF <50%	48%	0%	-	-	-
Sirico et al.	LVEF <55%	31%	11%	4%	-	-

On the initial follow-up echocardiography, five studies reported normal LVEF in all the patients they saw in clinic. In the other six studies, 2%–13% of the patients who came in for their first follow-up visit had ongoing depressed LVEF.

In the seven studies that showed second follow-up visit data, 3%–4% of patients showed ongoing depressed LVEF. Farooqui et al. showed that 3% of the patients who had decreased LVEF at their second follow-up had complete recovery of function at the third follow-up visit. Sirico et al. which showed 4% of patients with ongoing depressed LVEF does not have further follow-up data. Capone et al. and Chakraborty et al. notes persistent diastolic dysfunction in a small subset of patients (4%–9.5%) at their 6 month follow-up. All of these patients had LV systolic dysfunction during their hospitalization.

Table 3 summarizes the coronary artery dilation data from each study being reviewed. In terms of coronary artery dilation, three studies showed no coronary artery dilation in patients during admission. Of the other eight studies, there was coronary artery dilation in 9%–52% of patients during admission. There was complete resolution of coronary artery dilation in Farooqui et al. at the first follow-up visit. Others showed ongoing coronary artery dilation in 3%–28% of patients at the first follow-up. Barris et al., Jhaveri et al., and Arslan et al. showed ongoing coronary artery dilation in 6%, 17%, and 12% of patients respectively at the initial follow-up visit and did not have data on further follow-up visits. Four studies showed ongoing coronary artery dilation in 3%–12% of patients at the second follow-up visit. Capone et al. showed complete resolution of coronary artery dilation in all patients at the third follow-up visit. Omeir et al. showed ongoing coronary artery dilation in 4% of patients at the third follow-up visit 6 months later.

**Table 3:** Coronary artery dilation/aneurysm data.

	Definition	Admission	1st follow-up visit	2nd follow-up visit	3rd follow-up visit	4th follow-up visit
Farooqui et al.	*z*-score >2.0	9%	0%	0%	0%	-
Capone et al.	*z*-score >2.0	52%	28%	12%	0%	-
Das et al.	*z*-score >2.0	14%	3%	6%	-	-
Chakraborty et al.	*z*-score >2.0	0%	0%	0%	-	-
Arslan et al.	*z*-score >2.0	15%	12%	-	-	-
Barris et al.	*z*-score >2.0	19%	6%	-	-	-
Garbin et al.	*z*-score >2.0	0%	0%	0%	-	-
Jhaveri et al.	*z*-score >2.5	33%	17%	-	-	-
Omeir et al.	*z*-score >2.0	24%	11%	3%	4%	-
Patnaik et al.	*z*-score >2.0	0%	0%	-	-	-
Sirico et al.	*z*-score >2.0	28%	7%	9%	-	-

## Discussion

We reviewed eleven studies that report on follow-up visits and echocardiography findings in patients with MIS-C between 2020 and 2022. Due to the limited available data, we focused mainly on the timing of the follow-up visit, and LVEF and coronary artery dilation in the echocardiography reports.

### Timing of follow-up visits

**Figure 2:**
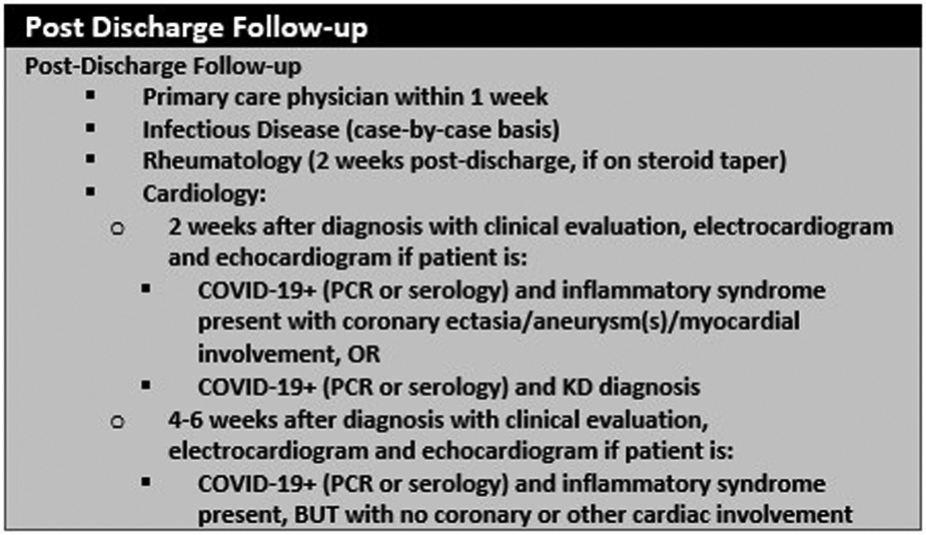
MIS-C follow-up guidelines at Lucile Packard Children's Hospital, Stanford University (17).

Since there are no standardized protocols for follow-up for children with MIS-C, many institutions have established their internal follow-up guidelines based on Kawasaki disease or myocarditis guidelines. Most studies we reviewed showed an initial cardiology visit within 1 month of discharge, which is similar to the 4–6 weeks recommendation for Kawasaki disease (16). However, unlike Kawasaki disease, there is no risk stratification for MIS-C either based on coronary artery dilation or the degree of ventricular dysfunction. Some institutions, such as ours (see Figure 2), have established follow up schedules based on hospital course and echocardiography findings during admission (1, 2, 17). For studies that had initial follow-up later than 1 month following discharge, it is unclear whether there were other medical visits (general pediatrician, rheumatology, infectious disease etc.) to continue to monitor for symptoms. However, follow-up with pediatric cardiology later than 1 month following discharge might delay evaluation of coronary arteries considering the risk for developing coronary artery dilation in the convalescent phase in patients with MIS-C. At our own institution, we recommend a follow-up with pediatric cardiology at 2 weeks after discharge if patients had coronary or myocardial involvement during admission and four to 6 weeks if patients had no coronary or myocardial involvement (17).

### Echocardiography findings

The findings of the studies reviewed here are similar to prior studies that have reported LVEF in MIS-C patients (4, 18, 19). There was depressed LVEF in ∼12%–62% of patients during admission in these studies. More than half the patients had normal left ventricular function as early as their initial follow-up echocardiography, which is comparable to myocarditis (20). However, mortality rate is low in MIS-C and there is no reported need for heart transplantation in pediatric population. There are several studies that look at follow-up CMR imaging in patients with LV dysfunction during admission which also shows normalization or improvement in LV function at the follow-up visit (21–23).

In terms of coronary artery dilation, most studies in this review showed 0%–30% of patients with coronary artery dilation with initial follow-up echocardiography within 1 month of discharge reassuringly showing resolution of coronary artery dilation in more than half of the patients. None of the studies note new onset of coronary artery dilation at the follow up visit. These data are important for outpatient pediatric cardiologists who are seeing patients with MIS-C as they are being evaluated for sports clearance and need for ongoing antiplatelet therapy or anticoagulation. Most institutions follow Kawasaki disease guidelines for patients with coronary artery involvement in MIS-C. While much about long-term course of MIS-C remains unknown, collective evaluation of single institutions is paramount to furthering our knowledge about this disease. There is also great anticipation for the MUSIC study which looks at the long term 5 year follow-up for patients with MIS-C to shed further light on this topic (24).

In terms of return to sports participation, only one of the studies reviewed commented on their practice which is to allow for clearance after normalization of inflammatory markers and systolic cardiac function usually around 8 weeks after hospital discharge (7).

### Limitations

This mini review is limited to only looking at the follow-up timelines and LV ejection fraction and coronary artery dilation amongst patients with MIS-C. We did not include data regarding laboratory values, valvular involvement, pericardial effusion, cardiac MRI imaging, or arrhythmias in this review. There are also limited information regarding the COVID-19 variants involved in each study.

## Conclusion

There is some institutional variation in the follow-up guidance for patients diagnosed with MIS-C. Based on limited publications to date, most institutions appear to have an initial follow-up visit within 1 month of discharge, however the further follow-up timelines vary. Follow-up guidelines based on the severity of initial presentation, hospital course, and echocardiographic findings are needed to provide guidance for physicians caring for patients with MIS-C.
